# Developing a comorbidity index for comparing cancer outcomes in Aboriginal and non-Aboriginal Australians

**DOI:** 10.1186/s12913-018-3603-y

**Published:** 2018-10-16

**Authors:** Lettie Pule, Elizabeth Buckley, Theophile Niyonsenga, David Banham, David Roder

**Affiliations:** 1 School of Health Sciences, Centre for Population Health Research, University of South Australia, Adelaide, SA 5001 Australia; 2 Centre for Research and Action in Public Health, University of Canberra, University Drive, Bruce, ACT 2617 Australia; 3 Wardliparingga Aboriginal Research Unit, South Australian Health and Medical Research Institute, North Terrace, Adelaide, SA 5000 Australia

**Keywords:** Comorbidity, Aboriginal, Cancer outcomes, Racial/ethnic disparity, Risk prediction, Cancer survival

## Abstract

**Background:**

Comorbidity is known to increase risk of death in cancer patients, both Aboriginal and non-Aboriginal. The means of measuring comorbidity to assess risk of death has not been studied in any depth in Aboriginal patients in Australia. In this study, conventional and customized comorbidity indices were used to investigate effects of comorbidity on cancer survival by Aboriginal status and to determine whether comorbidity explains survival disparities.

**Methods:**

A retrospective cohort study was undertaken using linked population-based South Australian Cancer Registry and hospital inpatient data for 777 Aboriginal people diagnosed with primary cancer between 1990 and 2010 and 777 randomly selected non-Aboriginal controls matched by sex, birth year, diagnosis year and tumour type. A customised comorbidity index was developed by examining associations of comorbid conditions with 1-year all-cause mortality within the Aboriginal and non-Aboriginal patient groups separately using Cox proportional hazard model, adjusting for age, stage, sex and primary site. The adjusted hazard ratios for comorbid conditions were used as weights for these conditions in index development. The comorbidity index score for combined analyses was the sum of the weights across the comorbid conditions for each case from the two groups.

**Results:**

The two most prevalent comorbidities in the Aboriginal cohort were “uncomplicated” hypertension (13.5%) and diabetes without complications (10.8%), yet in non-Aboriginal people, the comorbidities were “uncomplicated” hypertension (7.1%) and chronic obstructive pulmonary disease (4.4%). Higher comorbidity scores were associated with higher all-cause and cancer-specific mortality. The new index showed minor improvements in predictive ability and model fit when compared with three common generic comparison indices. After accounting for the competing risk of other deaths, stage at diagnosis, socioeconomic status, area remoteness and comorbidity, the increased risk of cancer death in Aboriginal people remained.

**Conclusions:**

Our new customised index performed at least as well, although not markedly better than the generic indices. We conclude that in broad terms, the generic indices are reasonably effective for adjusting for comorbidity when comparing survival outcomes by Aboriginal status. Irrespective of the index used, comorbidity has a negative impact on cancer-specific survival, but this does not fully explain the lower survival in Aboriginal patients.

## Background

Many cancer patients have concurrent chronic disease or conditions, commonly referred to as comorbidity. The risk of having comorbidity increases with age and can influence treatment choices [1–3]. Comorbidity has been shown to affect cancer diagnosis and treatment practices, and lead to an increased risk of hospitalisation, reduced quality of life, increased mortality, and potentially increased healthcare costs [3–5].

Comorbidity is an important determinant of poorer cancer outcomes [3], and potentially more so in Aboriginal than non-Aboriginal populations, due to a higher prevalence of comorbid conditions (e.g., diabetes, renal disease, injuries, diseases of the respiratory and cardiovascular systems, and infectious diseases) [6–10] .

Given the clinical importance of comorbidity and its high prevalence in ageing cancer populations, it is essential to have a measure for quantifying likely effects on cancer outcomes. Comorbidity indices have been developed to measure the extent of comorbid conditions and to quantify their impact on mortality [11–13]. These indices are often used for risk stratification or adjustment when comparing disease-specific survival or other health outcomes across different groups. The validity of these indices can vary, however, depending on the population, the diseases involved, the data sources and outcome measures [11–13].

Commonly used indices include the Charlson Comorbidity Index (CCI) and Elixhauser Comorbidity Index (ECI) [14, 15]. These indices were developed using general in-hospital patients to summarise risk of all-cause mortality at 1 year and in-hospital mortality due to comorbidity. Later, van Walraven developed weights for Elixhauser conditions using administrative data based on in-hospital mortality [16].

As the CCI and ECI were developed over two decades ago, and for limited purposes in general hospital patients, they may be sub-optimal indices of comorbidity for contemporary cancer populations. Previous studies have shown that ECI had a slightly better performance than CCI [13, 16]. Another population-based cancer specific index, which was developed based on the impact of comorbidity on non-cancer mortality, Cancer, Care and Comorbidity (C3), showed a superior model fit when compared to CCI [17]. Although cancer-specific, the C3 was not designed to meet the needs of an Australian Aboriginal cohort.

Due to the potential limitations of currently available comorbidity indices, and the lack of a gold standard method of measuring comorbidity [11–13]**,** this study was undertaken to: (1) describe the prevalence of comorbidities in Aboriginal and non-Aboriginal cancer populations in South Australia; and (2) develop a customised index for assessing the impact of this comorbidity on mortality by Aboriginal status. The utility of this index is demonstrated empirically by predicting excess mortality risk from comorbidity in a matched cohort and by comparing its predictive effects with that of generic indices. To the best of the authors’ knowledge, this is the first time a comorbidity index has been developed and customised for an Australian cancer cohort by Aboriginal status.

## Methods

### Data sources and linkage

Cancer data were extracted from South Australian Cancer Registry (SACR) and linked in-hospital data from all hospitals in SA, as incorporated in the South Australia Inpatient Hospital Separations (ISAAC) database. SACR is a population-based cancer registry that collects data on all cancers diagnosed in South Australia (excluding non-melanoma skin cancer). Notification of cancer is mandatory from pathology laboratories, radiotherapy centres, hospitals and the Registrar of Births, Deaths and Marriages (BDM). The SACR also collects mortality by routine linkage to South Australian death files and for deaths occurring outside of South Australia, to the National Death Index at the Australian Institute of Health and Welfare. Record linkage of data from the SACR, ISAAC, and BDM was conducted by SANT Data Link, with supplementary linkage to inpatient data undertaken by the SA Government Epidemiology Branch [18].

### Study population and variables

Due to small population numbers, Aboriginal and Torres Strait Islander people were combined and respectfully referred to as Aboriginal people in this study. All registered cancer cases in Aboriginal people diagnosed between 1990 and 2010 were included in this study. Aboriginal status of cases was checked through cross-referencing with other databases to reduce classification bias [18, 19]. Emphasis was placed on specificity at the expense of sensitivity to ensure that non-Aboriginal people were not misclassified as Aboriginal. Aboriginal cancer cases were paired with randomly selected control cancer cases in non-Aboriginal people, with matching for birth year, sex, primary cancer site and year of diagnosis**.** The matching process has been described in detail elsewhere [18]. The matched cohort was used for two purposes; firstly, to develop a general population cancer specific comorbidity index (PCSCI) with account taken of Aboriginal status, and secondly, to test the utility of the PCSCI for explaining survival differences between Aboriginal and non-Aboriginal people with cancer.

Demographic and clinical data were extracted from the SACR, including age at diagnosis (years), sex, primary site of cancer, tumour grade, degree of spread (local, regional, distant and unknown), and diagnostic period (1990–1999 or 2000–2010). Remoteness was derived from residential postcode at diagnosis [20], and indices of socioeconomic disadvantage measured by postcode categorised in quintiles (Q1: most disadvantage; Q5: least disadvantage) [21]. Death dates were included, plus causes of death coded as cancer or non-cancer by registry staff.

The International Classification of Diseases 10th Revision (ICD-10) was used to code comorbidity data obtained by extraction from patients’ in-hospital records for the 5 years preceding the primary cancer diagnosis. Comorbid conditions that were rarely reported in our population (prevalence not greater than 0.5%) were excluded. All cancer cases were followed-up from date of diagnosis to date of death, or to censoring on 31st December 2011, whichever came first. Patients with no record of death were assumed to be alive at the end of the follow-up period. For comorbidity index development, 1-year all-cause mortality was used as the outcome. For the validation of the index and subsequent survival analyses, both all-cause and cancer-specific mortality were outcomes of interest.

A Stata module was used to calculate CCI and ECI index scores based on originally developed CCI weights and modified to include ECI weights developed by van Walraven et al. [14–16, 22, 23]. Similarly, a SAS macro developed by Sarfati et al. [17] was converted into Stata and used to calculate C3 index score based on original weights developed for the 42 conditions in the all-sites C3 index. Scores for each participant were calculated separately for the respective indices as the sum of all the weights for the conditions recorded excluding cancers. The scores were treated as continuous variables for analysis and only categorised for descriptive purposes.

### Statistical analysis

Data were prepared using Stata version 14 (StataCorp, College Station, Texas) [24]. Initially an endeavour was made to rectify any missing data or errors by checking with data sources. Descriptive analyses of demographic and clinical characteristics were then made by Aboriginal status. Differences by Aboriginal status were tested using McNemar’s test for binary variables and Wilcoxon rank sum test for ordinal variables in the matched cohort.

### Development of a general population-based cancer-specific comorbidity index (PCSCI)

To develop a general PCSCI which can be applied in Aboriginal only, or combined Aboriginal and non-Aboriginal cohorts, the matched cohort was first split into Aboriginal and non-Aboriginal cases. Comorbidities were identified separately for each group. Cox proportional hazard models were fitted separately for each comorbid condition in the Aboriginal cohort and non-Aboriginal cohort respectively to avoid overfitting [17]. Models were adjusted for age, degree of spread (summary stage), sex and primary site, and the association of each comorbid condition with 1-year all-cause mortality determined. Adjusted hazard ratios (aHR) < 1 were assigned a “0” and those > = 1 were rounded to the nearest whole number. These aHRs were then assigned as weights for the respective Aboriginal and non-Aboriginal models. Weights thus obtained were summed across the two groups for each person to obtain a measure of comorbidity burden for the entire cohort (referred to as the PCSCI score) and used in subsequent analyses. Proportionality, a key Cox proportional hazards assumption, was assessed using Schoenfeld’s residuals and found to be met.

### Internal validation of the index

Bootstrapping was used to generate multiple samples from the matched cohort for validating and comparing the customised index performance with that of three pre-existing indices (two generic and one cancer specific). The performance of PCSCI, CCI, ECI and C3 indices, relative to baseline models for all-cause and cancer-specific mortality (adjusting for age and stage) was evaluated for discriminative ability using Harrell’s concordance statistic (C-index) using bootstrapping (1000 iterations) [25, 26], and the Akaike Information Criterion (AIC) for model fit using a stratified Cox regression model [27].

### The utility of PCSCI in explaining variation in survival

Models that included Aboriginal status were adjusted for remoteness, area level socioeconomic status (SES), degree of spread and respective measures of comorbidity (i.e., PCSCI, CCI, ECI and C3). Interactions between Aboriginality and remoteness, stage, age and comorbidity score, respectively were tested and included in the final model if approaching significance (*p* < 0.2). Additionally, multivariable regression models for competing risk analysis, using the Fine and Gray approach, were developed and used to assess the risk of death due to cancer from the estimated sub-hazard ratios (SHR) [28]. There was no meaningful difference between competing risk and disease-specific Cox proportional hazards regression, hence only competing risk results are presented in this report.

To assess the utility of the PCSCI for explaining differences in survival by Aboriginality, the percentage change in SHRs between restricted and unrestricted models were computed for each index as follows, and comparisons made across indices:

[(SHR _without comorbidity_ – SHR _comorbidity_)/ (SHR _without comorbidity_ − 1.0)] × 100

The study was reviewed and approved by the Human Research Ethics Committees of South Australian Health, the Aboriginal Health Council and University of South Australia. The Cancer Data and Aboriginal Disparities (CanDAD) project’s Aboriginal Community Reference Group (ACoRG) were consulted to ensure acceptability of the study to Aboriginal people and alignment with South Australian Aboriginal Health Research Accord principles [29].

## Results

A total of 777 Aboriginal primary cancer cases (mean age (standard deviation) = 57.7 years (± 15.6 years)), and 777 non-Aboriginal cases (mean age = 58.5 years (± 15.5 years)) were included in the matched cohort (Table 1). A higher proportion of Aboriginal were diagnosed at a distant stage (31.3% vs 22.0%), lived in outer regional and remote areas (50.3% vs 19.6%) and lived in low socioeconomic areas (55.5% vs 24.2%) compared with non-Aboriginal controls, (*p* < 0.05). By the end of follow-up period, 59.3% of Aboriginal people had died of cancer compared to 43.8% of non-Aboriginal controls (Table 1).

**Table 1:** Demographic and clinical characteristics of a matched South Australian Aboriginal and non-Aboriginal cohort diagnosed with cancer between 1990 and 2010

Demographic and clinical factors	Aboriginal	Non-Aboriginal	
	N (%)	N (%)	*p*-value
Age in years (mean, ± SD)	57.7 (± 15.6)	58.5 (± 15.5)	*p* = 0.232
Sex			
Male	375 (48.3)	375 (48.3)	
Female	402 (51.7)	402 (51.7)	*p* = 1.000
Decade of diagnosis			
1990–1999	284 (36.6)	284 (36.6)	
2000–2010	493 (63.5)	493 (63.5)	*p* = 1.000
Area-level socioeconomic status			
Q1 (Most disadvantaged)	431 (55.5)	188 (24.2)	
Q2	156 (20.1)	167 (21.5)	
Q3	76 (9.8)	129 (16.6)	
Q4	68 (8.8)	146 (18.8)	
Q5 (Least disadvantaged)	46 (5.9)	147 (18.9)	*p* < 0.001
Geographic remoteness			
Major cities	328 (42.2)	544 (70.0)	
Inner regional	58 (7.5)	81 (10.4)	
Outer regional	220 (28.3)	117 (15.1)	
Remote	171 (22.0)	35 (4.5)	*p* < 0.001
Degree of spread at diagnosis			
Localised	289 (37.2)	390 (50.2)	
Regional	155 (20.0)	132 (17.0)	
Distant	243 (31.3)	171 (22.0)	
Unknown/unstageable	90 (11.6)	84 (10.8)	*p* < 0.001
Grouped primary sites			
Head & neck	110 (14.2)	110 (14.2)	
Gastrointestinal	162 (20.9)	162 (20.9)	
Gynaecological	54 (7.0)	54 (7.0)	
Genitourinary	11 (1.4)	11 (1.4)	
Thoracic	124 (16.0)	124 (16.0)	
Breast	78 (10.0)	78 (10.0)	
Skin	34 (4.4)	34 (4.4)	
Lympho haematopoietic	39 (5.0)	39 (5.0)	
Prostate	45 (5.8)	45 (5.8)	
Unknown primary	46 (5.9)	46 (5.9)	
Other remaining sites	74 (9.5)	74 (9.5)	*p* = 1.000
Vital status at 31/12/2013			
Alive	220 (28.3)	349 (44.9)	
Non-cancer death	96 (12.4)	88 (11.3)	
Cancer death	461 (59.3)	340 (43.8)	*p* < 0.001

### Development of a general population-based cancer-specific comorbidity index (PCSCI)

Conditions included in the development of PCSCI and the numbers (%) of cancer cases affected are shown in Table 2. Hypertension (uncomplicated) and chronic obstructive pulmonary disease (COPD) were two of the most common comorbid conditions in both Aboriginal and non-Aboriginal cases, with a higher prevalence in Aboriginal compared to non-Aboriginal people (13.5% vs 7.1% for hypertension (uncomplicated) and 10.7% vs 4.4% for COPD) (Table 2). Although Aboriginal people had a higher comorbidity level than non-Aboriginal people, the assigned weights were generally lower for the same comorbid conditions than those assigned for non-Aboriginal people. PCSCI scores ranged from a low of 0 to a high of 25 for the matched cohort, with 29% having PCSCI score ≥ 1.

**Table 2:** Prevalence of comorbidities in South Australian population and association with 1-year all-cause mortality

Comorbidity	n (%)	Aboriginal aHR (95% CI)^a^	weight	n (%)	non-Aboriginal aHR (95% CI) ^a^	weight
Alcohol abuse	77 (9.9)	1.73 (1.25–2.39)	2	13 (1.7)	1.07 (0.44–2.64)	1
AMI	32 (4.1)	1.24 (0.75–2.04)	1	8 (1.0)	0.68 (0.21–2.18)	0
Angina	38 (4.9)	1.21 (0.78–1.89)	1	13 (1.7)	2.48 (1.06–5.82)	2
Anxiety and behavioural disorders	14 (1.8)	1.08 (0.44–2.66)	1	8 (1.0)	1.46 (0.35–6.01)	1
Blood loss anaemia	11 (1.4)	1.42 (0.52–3.88)	1	**	**	**
Bowel disease	18 (2.3)	1.10 (0.54–2.25)	1	5 (0.6)	11.17 (3.73–33.5)	11
Cardiac arrhythmias	30 (3.9)	0.80 (0.46–1.41)	0	22 (2.8)	0.99 (0.46–2.14)	0
CHF	36 (4.6)	1.48 (0.96–2.28)	1	14 (1.8)	2.09 (1.00–4.37)	2
Chronic viral hepatitis	18 (2.3)	0.97 (0.45–2.10)	0	**	**	**
Coagulopathy	18 (2.3)	1.06 (0.46–2.41)	1	9 (1.2)	1.36 (0.42–4.49)	1
COPD	83 (10.7)	1.13 (0.81–1.59)	1	34 (4.4)	1.64 (0.92–2.90)	2
CVD	26 (3.4)	1.33 (0.74–2.40)	1	15 (1.9)	2.12 (1.07–4.13)	2
Deficiency anaemia	22 (2.8)	1.07 (0.56–2.01)	1	14 (1.8)	0.81 (0.29–2.27)	0
Dementia	6 (0.8)	0.67 (0.16–2.72)	0	**	**	**
Depression	20 (2.6)	1.00 (0.49–2.04)	1	9 (1.2)	1.14 (0.28–4.64)	1
Diabetes complicated	58 (7.5)	1.13 (0.76–1.68)	1	6 (0.8)	1.27 (0.17–9.31)	1
Diabetes without complications	84 (10.8)	1.01 (0.72–1.42)	1	21 (2.7)	2.67 (1.39–5.13)	3
Drug abuse	29 (3.7)	0.97 (0.54–1.75)	0	6 (0.8)	2.34 (0.73–7.52)	2
Epilepsy	13 (1.7)	1.26 (0.55–2.88)	1	6 (0.8)	4.11 (1.27–13.3)	4
Eye problems	16 (2.1)	1.50 (0.83–2.70)	1	**	**	**
Fluid and electrolyte disorders	18 (2.3)	1.43 (0.75–2.71)	1	9 (1.2)	3.22 (1.40–7.38)	3
Hypertension uncomplicated	105 (13.5)	1.01 (0.74–1.38)	1	55 (7.1)	1.07 (0.63–1.80)	1
Hypothyroidism	12 (1.5)	1.77 (0.78–4.04)	2	**	**	**
Intestinal disorders	14 (1.8)	0.86 (0.35–2.12)	0	15 (1.9)	0.58 (0.18–1.85)	0
Joint and spinal disorders	5 (0.6)	2.62 (0.64–10.8)	3	**	**	**
Metabolic disorders	48 (6.2)	1.31 (0.88–1.95)	1	13 (1.7)	0.87 (0.32–2.36)	0
Mild liver disease	24 (3.1)	2.00 (1.17–3.40)	2	**	**	**
Moderate/Severe liver disease	**	**	**	6 (0.8)	3.23 (1.29–8.05)	3
Obesity	17 (2.2)	0.61 (0.22–1.64)	0	**	**	**
Osteoporosis	6 (0.8)	0.82 (0.26–2.59)	0	**	**	**
Other cardiac conditions	58 (7.5)	1.34 (0.92–1.95)	1	14 (1.8)	2.97 (1.35–6.56)	3
Other neurological disorders	11 (1.4)	0.90 (0.28–2.85)	0	**	**	**
Pancreatitis	6 (0.8)	0.80 (0.20–3.23)	0	**	**	**
Paralysis	13 (1.7)	1.89 (0.83–4.30)	2	9 (1.2)	2.31 (1.06–5.02)	2
Peptic ulcer	18 (2.3)	0.95 (0.46–1.94)	0	15 (1.9)	1.25 (0.51–3.09)	1
Psychoses	23 (3)	1.43 (0.75–2.72)	1	8 (1.0)	1.10 (0.27–4.46)	1
Pulmonary circulation disorders	7 (0.9)	0.79 (0.29–2.20)	0	**	**	**
PVD	6 (0.8)	0.23 (0.03–1.67)	0	6 (0.8)	1.23 (0.30–5.02)	1
Renal disease	34 (4.4)	2.37 (1.55–3.62)	2	5 (0.6)	8.56 (2.53–28.9)	9
Rheumatoid disease	7 (0.9)	1.77 (0.65–4.84)	2	6 (0.8)	1.77 (0.65–4.84)	2
Sleep disorders	5 (0.6)	1.91 (0.60–6.08)	2	**	**	**
Urinary tract disorders	7 (0.9)	2.19 (0.87–5.52)	2	**	**	**
Valvular diseases	13 (1.7)	1.48 (0.65–3.39)	1	6 (0.8)	1.46 (0.20–10.7)	1
Weight loss	17 (2.2)	2.97 (1.71–5.16)	3	7 (0.9)	1.21 (0.30–4.94)	1

Abbreviations: *AMI* acute myocardial infarction, *COPD* chronic obstructive pulmonary diseases, *CVD* cerebrovascular disease, *CHF* congestive heart failure

^a^adjusted for age, sex, stage, primary site

**excluded from the model, prevalence = < 0.5%

### Internal validation of the index

All indices showed similar predictive accuracy for all-cause and cancer-specific mortality (Table 3). The C-index ranged from 0.678 to 0.727 for all models using 1-year all-cause mortality, with PCSCI having the highest C-index score. Similarly, C-index score ranged from 0.689 to 0.733 for all models using 1-year cancer-specific mortality. Again, the highest C-index score applying for the PCSCI. The addition of the different comorbidity indices into the baseline model resulted in significant improvements in model fit, with PCSCI having a slightly lower AIC, indicating better model performance (Table 3).

**Table 3:** Internal validation and comparison of indices in predicting 1-year mortality in a matched cohort

	All-cause mortality		Cancer specific mortality	
Models	c-index	AIC	c-index	AIC
BL^a^	0.678	428	0.689	376
BL + PCSCI	**0.727**	**411**	**0.733**	**360**
BL+ CCI	0.716	413	0.724	362
BL + WECI	0.719	416	0.724	368
BL + C3	0.720	420	0.731	366

Abbreviations: *BL* baseline, *CCI* Charlson comorbidity index, *WECI* weighted Elixhauser comorbidity index, *C3* cancer care and comorbidity index, *PCSCI* Population cancer specific comorbidity index

^a^Baseline model adjusted for age, stage, stratified by (sex, year of birth, year of diagnosis and primary site)

^b^Bold entries represent best models

### The utility of PCSCI in explaining variation in survival

Unadjusted competing risk regression modelling showed that Aboriginal people had a higher risk of cancer death than their non-Aboriginal counterparts (SHR = 1.96, 95% CI; 1.71–2.24). After adjusting for stage, remoteness area, areal level SES and comorbidity, Aboriginal people still had elevated risk of cancer death, and this was significant for all indices, although there were not large differences in the reduction of death between indices (Table 4). There was a reduction in the risk of cancer-specific mortality after adjusting for area level SES, remoteness and stage, although the risk remained higher for Aboriginal people (SHR = 1.60, 95% CI; 1.34–1.92) (Table 4).

**Table 4:** Competing risk regression of factors associated with risks of cancer death, SA 1990–2010

Aboriginal – Yes	SHR (95% CI)
Unadjusted	1.96 (1.71–2.24)
Baseline model^a^	1.60 (1.34–1.92)
PCSCI	
Baseline model^a^	1.60 (1.34–1.92)
Adjusted for PCSCI	1.53 (1.28–1.83)
Proportion explained by comorbidity for the effect of Aboriginality on survival	12%
CCI	
Baseline model^a^	1.60 (1.34–1.92)
Baseline model^a^ + CCI	1.54 (1.28–1.84)
Proportion explained by comorbidity for the effect of Aboriginality on survival	10%
ECI	
Baseline model^a^	1.60 (1.34–1.92)
Adjusted for ECI	1.57 (1.31–1.87)
Proportion explained by comorbidity for the effect of Aboriginality on survival	5%
C3	
Baseline model^a^	1.60 (1.34–1.92)
Adjusted for C3	1.54 (1.29–1.85)
Proportion explained by comorbidity for the effect of Aboriginality on survival	10%

Abbreviations: *SHR* Sub hazard ratio, *CCI* Charlson comorbidity index, *WECI* weighted Elixhauser comorbidity index, *C3* cancer care and comorbidity index, *PCSCI* Population cancer specific comorbidity index

^a^Baselines model adjusted for stage, remoteness area and SES

The addition of comorbidity indices: CCI, WECI, C3, and PCSCI to the adjusted model reduced the SHR for Aboriginality by 10%, 5%, 10% and 12% respectively for cancer-specific mortality. Interactions between Aboriginality and area remoteness, and area level SES and age were not statistically significant. There was a significant interaction, however, between Aboriginality and comorbidity. This suggests that Aboriginal survival is modified disproportionately by comorbidity, with an increase in comorbidity score resulting in poorer survival.

## Discussion

As comorbidity is prevalent in cancer populations, and particularly in Aboriginal people, it is important when adjusting for its effects, that the best measure of comorbidity is used. In this study, there were slight differences in type of comorbidities between Aboriginal and non-Aboriginal people, with the prevalence generally being higher in Aboriginal people. The most common comorbid conditions found in this study for Aboriginal cases were the same as reported in previous studies [9, 10]. The two most common comorbid conditions in both populations were “uncomplicated” hypertension and COPD. This is a key finding for policy makers as it implies that proposed/planned interventions for these conditions would be applicable for both Aboriginal and non-Aboriginal cases.

Although Aboriginal people had a higher prevalence of comorbidity, the weights assigned to comorbid conditions were generally lower indicating that their impact on mortality was less severe than for non-Aboriginal cases. Also, weights assigned to comorbid conditions in the customised index of this study differed with those in three comparison indices, for the same comorbid conditions, potentially due to these conditions having a different mortality impact. Given the advances in treatment and management of chronic conditions, their contribution to the risk of mortality could have changed. This is consistent with a study by Quan et al. [30] which found reduced weights for some Charlson comorbid conditions. Furthermore, only 12 conditions were still predictive of mortality compared to 17 from the original CCI.

However, results need to be interpreted with caution as the matched non-Aboriginal cohort may not be representative of the South Australian non-Aboriginal population. Also, of note are the very large weights for bowel disease and renal failure in the non-Aboriginal cohort, which could be due to unstable estimates resulting from small numbers. By the end of 1-year follow up, all cases had died from all causes.

When weights were summed to get the overall PCSCI score, more people were identified as having a score greater than 0, compared to comparison indices. This indicates that generic indices can potentially underestimate the prevalence of comorbidity when applied in this population. This could be due to these indices including fewer conditions than the PCSCI; or alternatively, differences in comorbidity profiles of Aboriginal people compared to the general population. This finding further underscores the potential desirability of customised, population-specific comorbidity indices.

When PCSCI was validated and compared to other indices, however, results indicated only marginally better discrimination of the customised index than generic indices. These findings are nonetheless consistent with those of previous studies and suggestions that customized indices would provide more accurate measures and enable better adjustment for comorbidity [12, 13, 31]. Further improvements may be seen if individual comorbid conditions, rather than a summary measure, were used for adjustment in survival analyses.

When PCSCI was compared to a cancer-specific index, C3, it marginally outperformed it. One of the advantages of PCSCI over C3 is in its development where Aboriginality was taken into account when deriving weights for comorbidities. As such, PCSCI can be used flexibly for risk adjustments in both Aboriginal only and matched Aboriginal and non-Aboriginal cohorts. Also, incorporating Aboriginal status in weight development makes this index more representative of an Aboriginal cancer population, more accurate and with greater ability to adjust for comorbidity (as compared to C3 which was not developed with an Australian Aboriginal cohort in mind).

After accounting for degree of spread (summary stage), remoteness and socioeconomic status, the increased risk of cancer death in Aboriginal people remained. The addition of PCSCI and other indices led to a reduction of this disparity by between 5 and 12%, with a slightly higher reduction occurring with the PCSCI. Further investigations are needed to determine whether the differences in survival, which persisted after comorbidity adjustment, could be explained by other factors known to impact on survival but not included in the study, for example, lifestyle factors or adherence to treatment guidelines.

Lack of access to treatment data in this study limited our ability to investigate possible reasons for differences in mortality. Due to a small number of cases within each cancer site group, we could not test whether weights derived for each comorbid condition would differ by cancer type, which could influence overall comorbidity scores. Another limitation was not having access to Medicare data. It is envisaged that having access to Medicare data for this study population could potentially strengthen our index, as more comorbid conditions which might impact on mortality risk could be inferred and added to the patient’s total score (including conditions not associated with hospitalisation).

The greatest strengths of this study were accessing data where Aboriginal status was cross-validated and were the sample of cases was drawn from the entire Aboriginal cohort diagnosed with cancer in South Australia for the period under review, thus minimising the potential for selection bias. The PCSCI was developed in South Australia, and it is recommended that PCSCI be validated in an external Aboriginal population to assess its generalizability to Aboriginal populations in other Australian states. The availability of comparable data for linking at a population level throughout Australia underscores the usefulness of data linkage to inform policy and to provide evidence of risk factors which impact on cancer outcomes.

In conclusion, our study is the first to develop a comorbidity index specific to cancer population of South Australia which can be applied effectively to Aboriginal only or a combined population. The results showed that PCSCI performed as well as or marginally better than CCI, ECI and C3. Nonetheless results also indicate that generic comorbidity indices developed in other non-Aboriginal populations probably provide a reasonably accurate determinations of comorbidity effects in Aboriginal people with cancer.

## Abbreviations

ABS: Australian Bureau of Statistics
aHR: Adjusted hazard ration
AIC: Akaike information criterion
ARIA: Accessibility/Remoteness Index of Australia
CCI: Charlson Comorbidity Index
CI: Confidence interval
COPD: Chronic obstructive pulmonary diseases
ECI: Elixhauser Comorbidity Index
ICD-10: International classification of diseases tenth revision
IRSD: Index of relative socio-economic disadvantage
ISAAC: South Australia Inpatient Hospital Separations
NDI: National Death Index
SA: South Australia
SACR: South Australian Cancer Registry
SEIFA: Socio-economic index for areas
